# Hyperreflective material in patients with non-neovascular pachychoroid disease

**DOI:** 10.1186/s12886-023-03011-2

**Published:** 2023-06-06

**Authors:** Maiko Maruyama-Inoue, Yasuo Yanagi, Shaheeda Mohamed, Tatsuya Inoue, Yoko Kitajima, Shoko Ikeda, Kazuaki Kadonosono

**Affiliations:** 1 Department of Ophthalmology and Micro-Technology, Yokohama City University, 4–57 Urafune-cho, Minami-ku, Kanagawa 232 − 0024 Yokohama, Japan; 2 Department of Ophthalmology and Visual Sciences, Hong Kong Eye Hospital, The Chinese University of Hong Kong, Hong Kong Special Administrative Region, Hong Kong, China

**Keywords:** Pachychoroid, Hyperreflective foci, Subretinal hyperreflective material, Macular neovascularization, Age-related macular degeneration, Idiopathic choroidal neovascularization, Pachychoroid pigment epitheliopathy, Drusen ooze, Focal choroidal excavation

## Abstract

**Background:**

This study aimed to report eleven cases of non-neovascular pachychoroid disease with hyperreflective material (HRM) that occurred in Japanese patients.

**Methods:**

A retrospective review of data from eleven patients who had non-neovascular retinal pigment epithelium (RPE) protrusion with HRM in the neurosensory retina between March 2017 and June 2022 was conducted. Clinical examination, color fundus photography, fluorescein angiography, spectral-domain optical coherence tomography (SD-OCT), and OCT angiography data were analyzed. Main outcome measures were patient characteristics, changes in SD-OCT findings, and symptom outcomes.

**Results:**

All cases had RPE protrusion and HRM with dilated choroidal veins, which were characteristic of pachychoroid disease. However, none of the cases had macular neovascularization (MNV). In 9 eyes (81.8%), HRM improved spontaneously without intervention and resulted in alterations in RPE, referred to as pachychoroid pigment epitheliopathy (PPE) or focal choroidal excavation (FCE). In these cases, symptoms such as metamorphopsia and distortion improved without treatment. In the remaining two cases (18.2%), HRM still persisted during the follow-up period.

**Conclusion:**

There are some cases of non-neovascular pachychoroid disorder with HRM, which might be a new entity of pachychoroid spectrum disease or an early stage of PPE or FCE. These cases should not be misdiagnosed as MNV, and careful observation is necessary.

## Background

The development of optical coherence tomography (OCT) [1] and OCT angiography (OCTA) [2], which are novel noninvasive multimodal imaging modalities, has enabled the evaluation of the choroidal structure and morphology in patients with various chorioretinal diseases. Using these modalities, pachychoroid spectrum diseases, characterized by thick choroid, pachyvessels, attenuation of the inner choroid, and choroidal vascular hyperpermeability on indocyanine green angiography (ICGA), have been recently described [3]. Pachychoroid neovasculopathy [4] and polypoidal choroidal vasculopathy [5] are included in the pachychoroid-related spectrum of diseases associated with type 1 macular neovascularization (MNV). Furthermore, pachychoroid pigment epitheliopathy (PPE) [6], focal choroidal excavation (FCE) [7] and central serous chorioretinopathy (CSC) [8] also reside within the category of pachychoroid-related diseases with non-neovascular lesions. However, previous cases associated with non-neovascular pahychoroid disease showed no hyperreflective material (HRM) in the neurosensory retina.

Warrow et al. reported that PPE should be suspected in eyes with choroidal thickening and associated abnormalities of the retinal pigment epithelium (RPE) with neither history of subretinal fluid (SRF) nor type 1 MNV [6]. On the other hand, Jampol et al. firstly described that FCE is characterized by an unusual concavity in the choroid without staphyloma or scleral ectasia [9] and occurs in combined with other macular diseases such as CSC or MNV [10]. However, all cases reported in their articles had no HRM in the neurosensory retina. Recently, our group experienced cases of non-neovascular pachychoroid disease with HRM among relatively young Japanese patients, which finally resulted in PPE or FCE after the resolution of HRM. The purpose of this study is to put together these cases that describe that entity and provides a natural history for the HRM.

## Methods

We conducted a retrospective review of the data of patients who had non-neovascular pachychoroid lesions with HRM in the neurosensory retina between March 2017 and June 2022. We studied eleven eyes of eleven patients in an observational case series. The patients were seen at Yokohama City University Medical Center after undergoing a prior evaluation by outside ophthalmologists, or were diagnosed by retina specialists (M.M. and Y.Y.) in a private clinic for metamorphopsia or visual complaints. This study design was approved by the institutional review board at Yokohama City University Medical Center and followed the tenets of the Declaration of Helsinki.

Inclusion criteria were the presence of non-neovascular pachychoroid lesions with HRM in the neurosensory retina determined by clinical findings and multimodal imaging. HRM were defined as hyper- or isoreflective exudation by using spectral-domain OCT (SD-OCT, SPECTRALIS OCT2 Multicolor version 6.12; Heidelberg Engineering Inc., Dossenheim, Germany and RS-3000 Advance; Nidek Co., Ltd., Aichi, Japan). SD-OCT system with built-in eye-tracking can provide reproducible measurements and we were able to evaluate the same location on OCT images at different time point [11]. Fluorescein angiography (FA), ICGA (SPECTRALIS Product Family version 5.3; Heidelberg Engineering, Inc.), fundus autofluorescence (FAF; Optos California®, Optos PLC, Dunfermline, UK), and OCTA (DRI OCT Triton; Topcon Corporation, Tokyo, Japan) were also used to evaluate the HRM lesions.

Central choroidal thickness (CCT) was measured in all eyes and was defined as the maximum thickness between the Bruch’s membrane and the inner surface of the choroidal–scleral junction at the fovea. Manual digital calipers were used to acquire these measurements. These cases were collected via a chart review, and data from these patients were reviewed. Changes in SD-OCT findings, changes of best-collected visual acuity (BCVA), and outcomes of the patients’ symptoms were investigated. Comparison of BCVA and CCT between baseline and last visit was performed using the Wilcoxon signed-rank test.

## Results

**Fig. 1:**
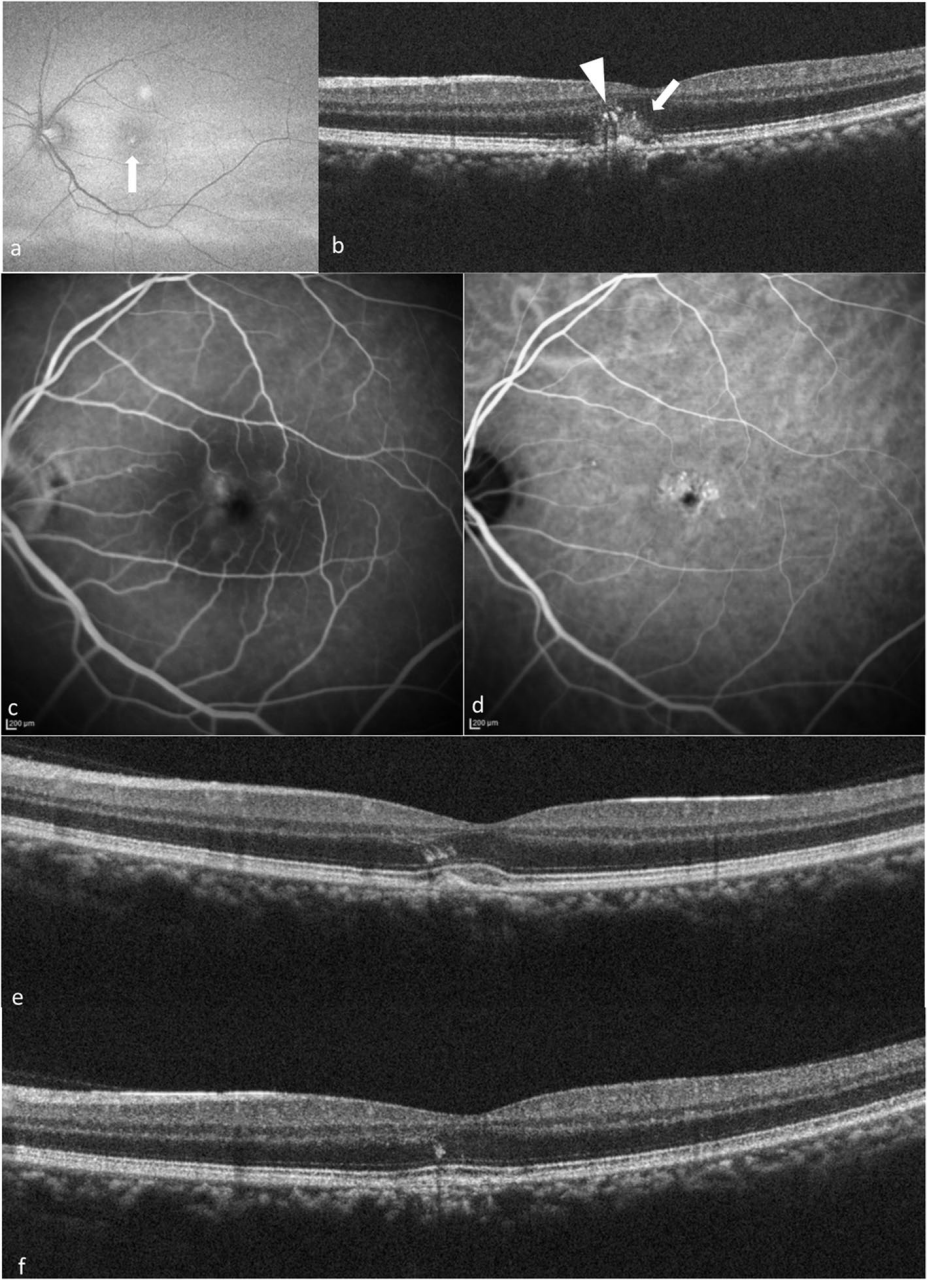
Multimodal imaging of case 1. **a** A 47-year-old man presented to a private clinic with visual complaints in his left eye. On examination, BCVA was found to be 20/20 in the right eye and 20/30 in the left eye. Dilated funduscopic examination showed a white lesion in the macula, which appeared hyperautofluorescence on FAF (arrow). **b** SD-OCT showed slight protrusion of RPE with HRM. The hyperreflective dots at the ONL and OPL (arrowhead) and the isoreflective exudation at the subretina and ONL (arrow) were seen. CCT was measured as 349 μm. The choroid had several dilated choroidal veins. **c** FA showed hypofluorescent with surrounding slight hyperfluorescence. **d** ICGA showed hypofluorescence without MNV. **e** This patient was observed without intervention, and HRM improved spontaneously in 6 months although presence of hyperreflective dots were noted at the ONL. His BCVA improved to 20/20. **f** Although hyperreflective dots still persisted at the ONL, HRM almost disappeared and the elevation of RPE also improved

**Fig. 2:**
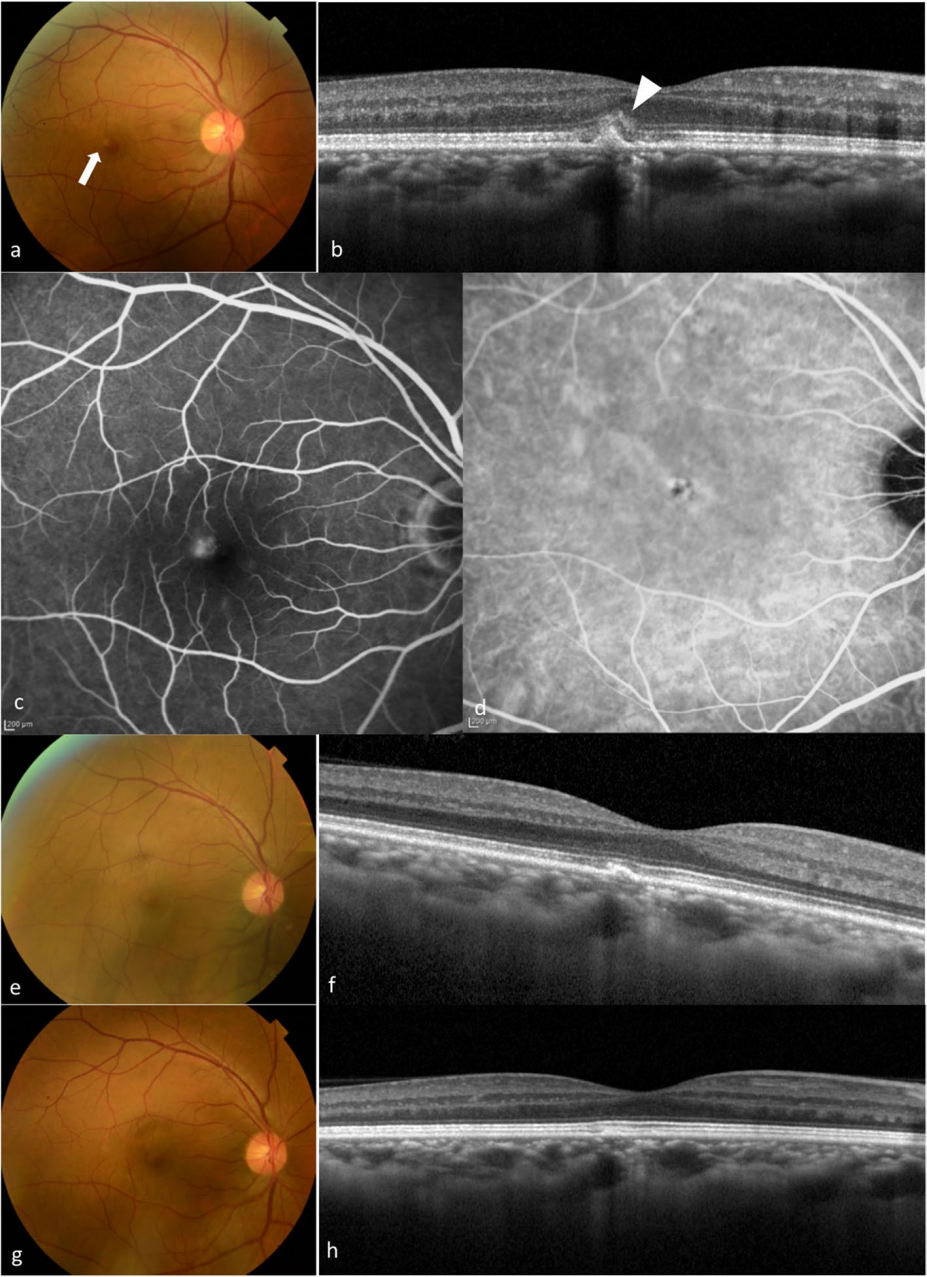
Multimodal imaging of case 2. **a** A 44-year-old man presented with distortion in his right eye and was referred for suspected ICNV. On examination, BCVA was found to be 20/16 in both eyes. The results of funduscopic examination in the patient’s right eye demonstrated a white lesion in the macula (arrow). **b** SD-OCT demonstrated a CCT of 268 μm with pachyvessels and elevation of RPE with slight HRM at subretina and the ONL (arrowhead). **c** FA showed staining corresponding to the white lesion. **d** ICGA showed hypofluorescence without MNV. **e** The patient was also observed without intervention, and the white lesion improved spontaneously after 6 months. His BCVA was maintained at 20/16. **f** SD-OCT at 6 months showed that the HRM had disappeared and the elevation of RPE also improved. **g** At 1 year, BCVA was maintained at 20/16 without recurrence. **h** SD-OCT presented almost normal RPE

**Fig. 3:**
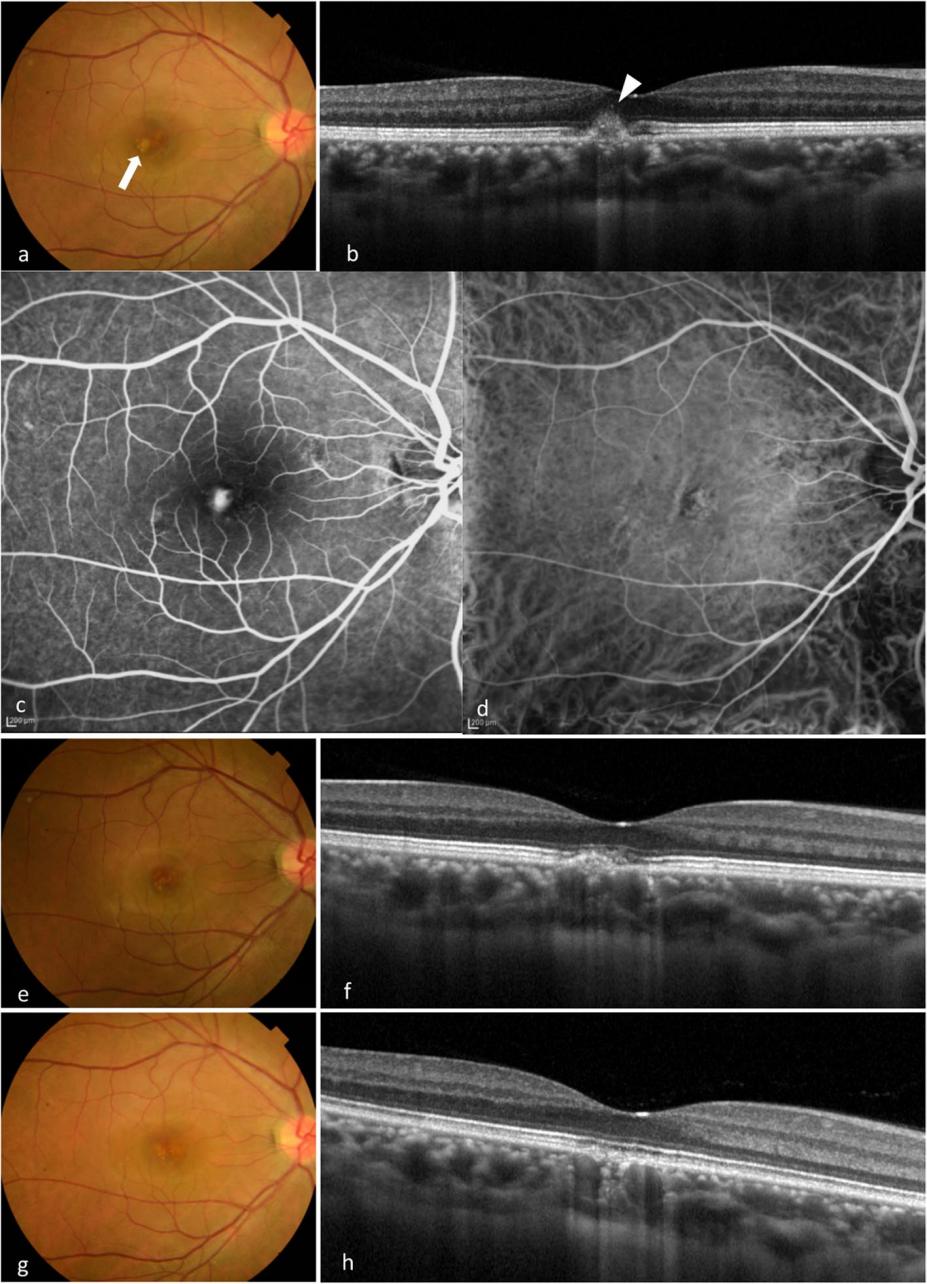
Multimodal imaging of case 3. **a** A 36-year-old woman presented with visual complaints in her right eye. She was referred from a private clinic as macular atrophy. BCVA was found to be 20/25 in the right eye and 20/16 in the left eye. Examination revealed a white lesion in the macula (arrow). **b** SD-OCT revealed a CCT of 305 μm in the right eye with pachyvessels. Elevation of RPE with HRM at the subretina was also observed. **c** FA showed hyperfluorescence corresponding to the white lesion. **d** ICGA also demonstrated hyperfluorescence, although there was no MNV. **e** The white lesion had almost disappeared 1 month later. **f** HRM at the subretina also disappeared on SD-OCT, although the elevation of RPE remained. **g** After 6 months, the patient’s BCVA improved to 20/16. **h** SD-OCT showed RPE alterations in the macula

**Fig. 4:**
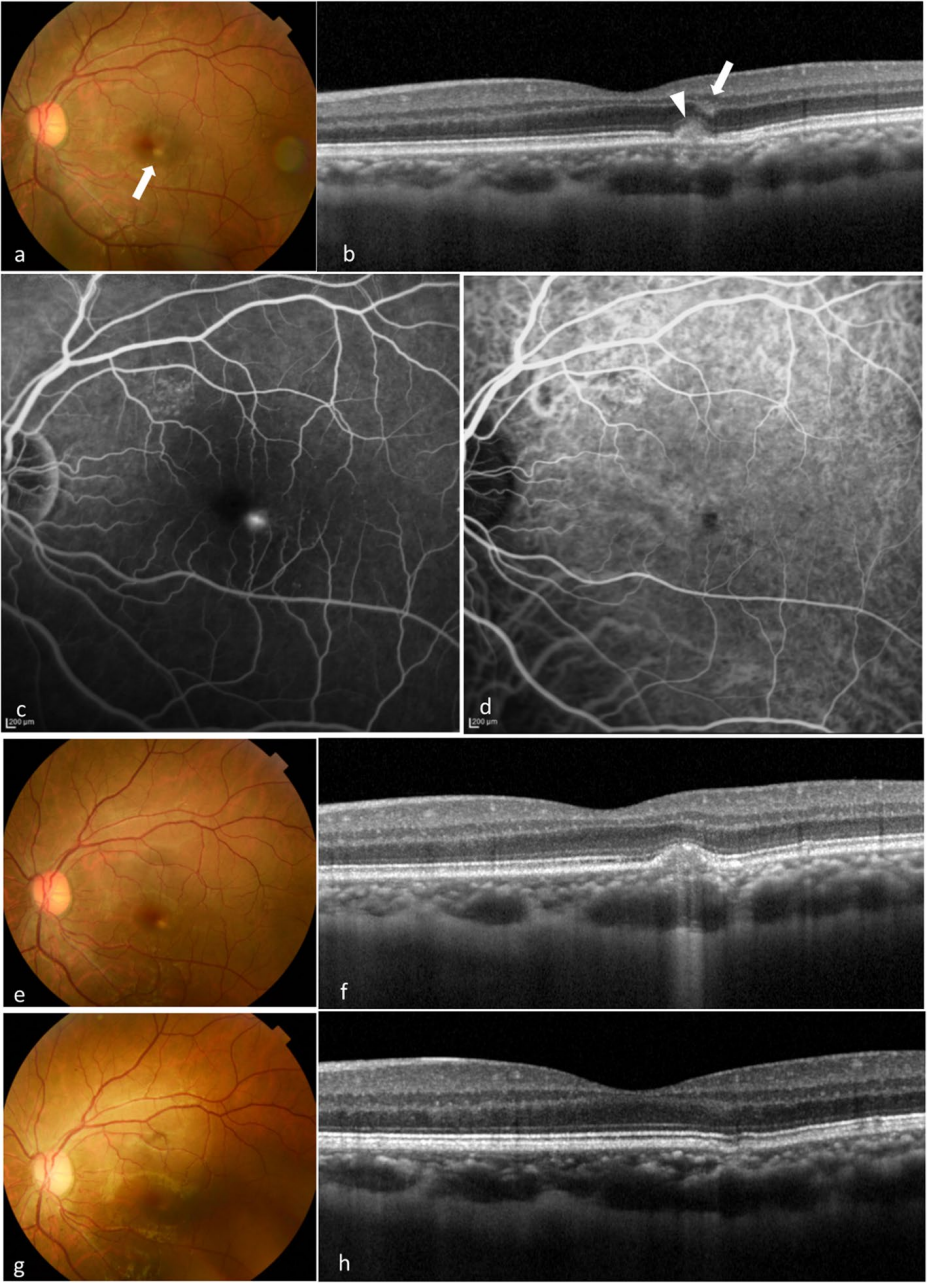
Multimodal imaging of case 5. **a** A 30-year-old man presented with metamorphopsia in his left eye. He was referred from a private clinic to our hospital. His BCVA was found to be 20/20 in both eyes. Examination demonstrated a white lesion in the macula (arrow). **b** SD-OCT revealed CCT of 253 μm in the left eye with pachyvessels. RPE changes with HRM at the subretina (arrowhead), OPL and ONL (arrow) were also observed. **c** FA showed hyperfluorescence corresponding to the white lesion. **d** ICGA demonstrated an area of hypofluorescence, which showed no MNV. **e** After 1 month, the white lesion remained. **f** However, SD-OCT demonstrated that HRM almost disappeared. **g** After 6 months, the white lesion disappeared, and the patient’s symptoms also improved. **h** SD-OCT showed FCE

**Fig. 5:**
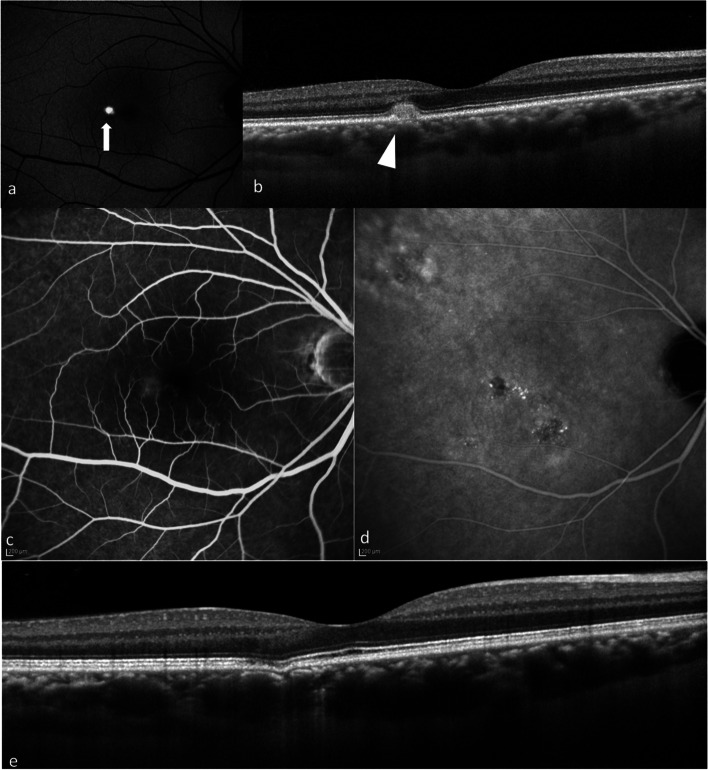
Multimodal imaging of case 9. **a** A 39-year-old man was referred from a private clinic with abnormality in OCT in his right eye. His BCVA was found to be 20/16 in the right eye. FAF showed hyperautofluorescence corresponding to the lesion (arrow). **b** SD-OCT revealed protrusion of RPE with HRM at the subretina. CCT was 346 μm in the right eye with dilated choroidal vessels. **c** FA showed staining corresponding to the white lesion. **d** ICGA demonstrated an area of hypofluorescence, which showed no MNV. **e** After 3 months, SD-OCT demonstrated that HRM improved spontaneously, however, FCE presented

We included a total of eleven eyes from eleven patients in this study. Table 1 shows the demographic and clinical characteristics of patients of non-neovascular pachychoroid disease with HRM in the neurosensory retina. Two of the eleven patients were given a diagnosis of idiopathic choroidal neovascularization (ICNV) or age-related macular degeneration (AMD) from their previous ophthalmologists, and two patients were referred for RPE changes in the macula. Of the eleven patients, eight (72.7%) were men and three (27.3%) were women. The mean patient age was 40.8 ± 10.7 years (median, 43 years; range, 23–60 years). The mean CCT was 307 ± 62 μm (range, 227–440 μm). All eleven eyes demonstrated pathologically dilated choroidal vessels (pachyvessels) on SD-OCT. The mean logarithm of the minimum angle of resolution (logMAR) BCVA was 0.062 (Snellen 20/23, range; 20/40 − 20/16). Representative cases of the patients are shown in Figs. 1, 2, 3, 4 and 5, respectively.

**Table 1:** Baseline characteristics and clinical data of the patients

Case	Patients	R/L	Age	Gender	Choroidal thickness(µm)		Best-corrected Visual Acuity		Final HRM	Location of HRM	Acoustic hyperreflectivity	PPE at final	FCE at final
					Baseline	Last visit	Baseline	Last visit					
**1**	1	L	47	M	349	324	20/30	20/20	improved	Subretina, ONL, OPL	+	+	-
**2**	2	R	44	M	268	299	20/16	20/16	improved	Subretina, ONL	-	-	-
**3**	3	R	36	F	305	290	20/25	20/16	improved	Subretina	-	+	-
**4**	4	R	43	M	314	316	20/32	20/40	improved	Subretina	+	+	-
**5**	5	L	30	M	253	263	20/20	20/20	improved	Subretina, ONL, OPL	+	-	+
**6**	6	R	60	M	351	362	20/25	20/25	persist	ONL, OPL	+	-	-
**7**	7	R	45	F	440	391	20/25	20/25	persist	Subretina, ONL, OPL	-	-	-
**8**	8	R	52	M	248	261	20/25	20/20	improved	Subretina	-	+	-
**9**	9	R	39	M	346	341	20/16	20/16	improved	Subretina	-	-	+
**10**	10	R	23	M	279	269	20/40	20/16	improved	Subretina, ONL	-	-	-
**11**	11	R	30	F	227	237	20/20	20/16	improved	Subretina	-	-	+

*HRM* Hyperreflective material, *PPE* Pachychoroid pigment epitheliopathy, *FCE* Focal choroidal excavation, *ONL* Outer nuclear layer, *OPL* Outer plexiform layer

### Changes in SD-OCT findings

Of these 11 eyes, HRM at the subretinal space were present in 10 eyes (90.9%). There was presence of HRM at the outer nuclear layer (ONL) in 6 eyes (54.5%) and at the outer plexiform layer (OPL) in 4 eyes (36.4%). Among these 11 patients, 4 eyes (36.4%) had acoustic hyperreflectivity behind the HRM. In nine patients (81.8%), HRM improved spontaneously without intervention such as anti-vascular endothelial growth factor (VEGF) or anti-inflammatory therapy within 1 year. Four of nine (44.4%) eyes resulted in RPE alterations, which is referred to as PPE. Furthermore, three of nine eyes (33.3%) showed focal choroidal excavation (FCE) after the improvement of HRM. On the other hand, HRM persisted during the follow-up period in the remaining two eyes (18.2%). The mean CCT in last visit was 305 ± 47 μm, which was no significant difference compared with baseline (*p* = 0.929).

### Symptom outcomes

The mean log MAR BCVA significantly improved from 0.062 to -0.001 (Snellen 20/19) (*p* = 0.046). Symptoms such as metamorphopsia or distortion also improved in nine eyes (81.8%) where the HRM ameliorated. On the other hand, the remaining two eyes (18.2%) with persistence of HRM showed no improvement of their symptoms.

## Discussion

Our observational case series demonstrated that there are some cases of non-neovascular disease with HRM. All cases had thickened choroids and pachyvessels at a relatively young age. Furthermore, HRM improved spontaneously without intervention in nine eyes (81.8%) and finally resulted in so-called PPE or FCE. Although these cases are very rare in a real-world clinical setting, it could be a new entity of pachychoroid spectrum disease or an early stage of PPE or FCE. In addition, these cases might be misdiagnosed as AMD or ICNV because of the existence of HRM and suspected MNV. However, it is important that these cases should be diagnosed as non-neovascular disease using multimodal imaging and carefully observed without any intervention. To our knowledge, non-neovascular pachychoroid disorder with HRM reported in this study has not previously been described.

In the previous studies, HRM at the subretinal space were reported as subretinal hyperreflective material (SHRM). In the Comparison of Age-Related Macular degeneration Treatments Trials (CATT), SHRM was an important morphologic biomarker for worse visual acuity in patients with AMD [12]. SHRM is known to have several components, such as neovascular tissue, fibrosis, hemorrhage, and exudation, likely comprising serum, fibrin, and inflammatory cells [13–15]. However, in our cases, it is deniable that HRM consists of neovascular tissue, fibrosis, or hemorrhage based on fundoscopic findings or multimodal imaging. Therefore, in this study, it is highly possible that HRM components were some kind of exudation. A previous study reported that in patients with CSC, which is categorized as one type of pachychoroid spectrum disease, breaches in the RPE could allow a large molecule, such as fibrin, to enter from the sub-RPE into the subretinal spaces [16]. Although the reason why patients in our study demonstrated HRM is uncertain, damage to the choriocapillaris, which was caused by the pachyvessels [17], would allow a large molecule such as fibrin to escape into the sub-RPE space. Furthermore, damage to the RPE would allow this molecule to enter from the sub-RPE to the subretinal spaces or neurosensory retina.

In our study, HRM were seen not only at the subretinal space but also at the OPL and ONL. Previous studies described that hyperreflective foci (HRF) [18], which is defined as hyperreflective speckling in the neurosensory retina detected by using SD-OCT, have been identified in various chorioretinal diseases [19–21]. In this study, 4 eyes (case 1, 4, 5, and 6) had HRF, and these cases had acoustic hyperreflectivity behind the HRM. Proposed origin of HRF have several hypotheses, such as displacing RPE cells [18], activated microglia cells [22], and lipid extravasation [23]. Therefore, in some cases of this study, HRM might consist of these components.

In nine cases (81.8%), HRM improved spontaneously and four of them resulted in so-called PPE and three of them showed FCE. Symptoms such as metamorphopsia or distortion also improved in these eyes where the HRM ameliorated. Because both PPE and FCE are usually asymptomatic, we speculate that HRM was the cause of their symptoms. A previous study reported the morphologic characteristics of patients with CSC evaluated by SD-OCT [24]. Those authors described that SRF with subfoveal fibrin was more commonly observed in the early period of acute CSC than in eyes with chronic CSC. We speculate that having HRM indicates an active stage, and these characteristics disappear spontaneously when the activity declines. In addition, RPE alterations occur after improvement of HRM. Monés et al. described that some patients with soft drusen due to AMD showed isoreflective dots at the outer retinal layers associated with RPE defects. They have termed this ‘drusen ooze’, which was associated with a high risk of developing atrophy [25]. They hypothesized that drusen ooze could activate the surface of RPE, resulting in an increase in phagocytosis of extracellular debris that exceeds the RPE capacity, and finally leads to RPE death. Morphologic characteristics in our study are similar to that of their study. Therefore, HRM may activate the RPE apical surfaces, phagocytosed by RPE cells, and finally morphologic features might be similar to PPE.

Furthermore, three eyes (27.3%) showed FCE after the resolution of HRM. Although the pathogenesis of FCE formation is still uncertain, previous reports described that FCE lesions are congenital choroidal abnormalities [9, 26]. On the other hand, some reports mentioned that FCE may occur in associated with inflammatory diseases such as multiple evanescent white dot syndrome, multifocal choroiditis, or Vogt–Koyanagi–Harada disease [27–29]. Similar to these cases, HRM in the neurosensory retina, due to inflammatory process, could be a mechanism leading to FCE formation. However, all cases in our study didn’t need anti-inflammatory therapy and improved spontaneously. Inflammation caused by HRM may be weak enough not to require treatment. Otherwise, it is a self-limited disorder.

In this study, the differential diagnosis was neovascular diseases, such as ICNV and AMD, because SHRM, which is derived from exudation, is generally associated with active MNV and often regresses as the MNV becomes quiescent after anti-VEGF treatment [14, 30]. However, all cases in this study had no MNV by multimodal imaging and were finally observed carefully without any intervention. Because the current optimal treatment for either ICNV or AMD is anti-VEGF agents [31, 32], it is important to check the existence of MNV by multimodal imaging and differentiate non-neovascular lesions with HRM from ICNV or AMD.

**Fig. 6:**
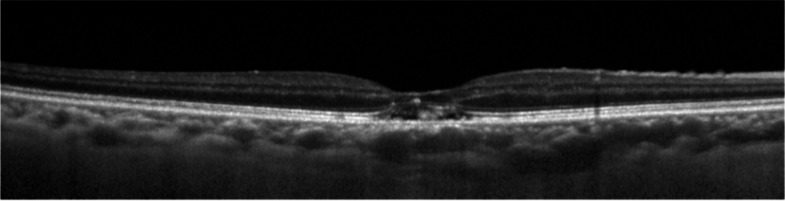
A case of non-neovascular pachychoroid disease with HRM and SRF. SD-OCT showed slight SRF with HRM at the subretinal space

There are also some cases of non-neovascular pachychoroid lesion with HRM, which have SRF, as shown in Fig. 6. We speculate that the pathological mechanism of these cases are similar to our cases without SRF. However, these cases are now generally categorized as CSC due to SRF and photodynamic therapy might be taken into consideration for treatment [33].

The limitations of this study are the small sample size and its retrospective nature. Although these cases are very rare in the clinical setting, larger numbers of study subjects with detailed analysis are needed in the future. In addition, the evaluation of multimodal imaging could be reader dependent; to minimize this confounding factor, an AI machine system that includes machine-learning algorithms should be investigated. This could enable earlier detection of non-neovascular pachychoroid diseases and demonstrate there is no need for treatment.

## Conclusions

We experienced eleven cases that had non-neovascular pachychoroid diseases with HRM. In addition, it is important to avoid a misdiagnosis of ICNV or AMD. It should be noted that non-neovascular pachychoroid diseases with HRM, occurring in relatively young adults, improve spontaneously without intervention.

## Data Availability

The datasets used during the current study are available from the corresponding author on reasonable request.

## Abbreviations

OCT: Optical coherence tomography
OCTA: OCT angiography
ICGA: Indocyanine green angiography
MNV: Macular neovascularization
PPE: Pachychoroid pigment epitheliopathy
FCE: Focal choroidal excavation
CSC: Central serous chorioretinopathy
HRM: Hyperreflective material
RPE: Retinal pigment epithelium
SRF: Subretinal fluid
SD-OCT: Spectral-domain OCT
FA: Fluorescein angiography
FAF: Fundus autofluorescence
CCT: Central choroidal thickness
BCVA: Best-corrected visual acuity
ICNV: Idiopathic choroidal neovascularization
AMD: Age-related macular degeneration
Log MAR: Logarithm of the minimum angle of resolution
ONL: Outer nuclear layer
OPL: Outer plexiform layer
VEGF: Vascular endothelial growth factor
SHRM: Subretinal hyperreflective material
HRF: Hyperreflective foci

## References

[1] Spaide RF, Koizumi H, Pozzoni MC (2008). Enhanced depth imaging spectral-domain optical coherence tomography. Am J Ophthalmol.

[2] Spaide RF, Klancnik JM, Cooney MJ (2015). Retinal vascular layers imaged by fluorescein angiography and optical coherence tomography angiography. JAMA Ophthalmol.

[3] Cheung CMG, Lee WK, Koizumi H, Dansingani K, Lai TYY, Freund KB (2019). Pachychoroid disease. Eye (Lond).

[4] Pang CE, Freund KB (2015). Pachychoroid neovasculopathy. Retina.

[5] Balaratnasingam C, Lee WK, Koizumi H, Dansingani K, Inoue M, Freund KB (2016). Polypoidal Choroidal Vasculopathy: a distinct disease or manifestation of many?. Retina.

[6] Warrow DJ, Hoang QV, Freund KB (2013). Pachychoroid pigment epitheliopathy. Retina.

[7] Chung H, Byeon SH, Freund KB, Focal choroidal excavation and its association with pachychoroid spectrum disorders (2017). A review of the literature and Multimodal Imaging Findings. Retina.

[8] Fung AT, Yannuzzi LA, Freund KB (2012). Type 1 (sub-retinal pigment epithelial) neovascularization in central serous chorioretinopathy masquerading as neovascular age-related macular degeneration. Retina.

[9] Jampol LM, Shankle J, Schroeder R, Tornambe P, Spaide RF, Hee MR (2006). Diagnostic and therapeutic challenges. Retina.

[10] Obata R, Takahashi H, Ueta T, Yuda K, Kure K, Yanagi Y (2013). Tomographic and angiographic characteristics of eyes with macular focal choroidal excavation. Retina.

[11] Menke MN, Dabov S, Knecht P, Sturm V (2009). Reproducibility of retinal thickness measurements in healthy subjects using spectralis optical coherence tomography. Am J Ophthalmol.

[12] Willoughby AS, Ying GS, Toth CA, Maguire MG, Burns RE, Grunwald JE, Daniel E, Jaffe GJ (2015). Comparison of age-related Macular Degeneration treatments trials Research G: Subretinal Hyperreflective Material in the comparison of age-related Macular Degeneration treatments trials. Ophthalmology.

[13] Ristau T, Keane PA, Walsh AC, Engin A, Mokwa N, Kirchhof B, Sadda SR, Liakopoulos S (2014). Relationship between visual acuity and spectral domain optical coherence tomography retinal parameters in neovascular age-related macular degeneration. Ophthalmologica.

[14] Ores R, Puche N, Querques G, Blanco-Garavito R, Merle B, Coscas G, Oubraham H, Semoun O, Souied EH (2014). Gray hyper-reflective subretinal exudative lesions in exudative age-related macular degeneration. Am J Ophthalmol.

[15] Spaide RF, Jaffe GJ, Sarraf D, Freund KB, Sadda SR, Staurenghi G, Waheed NK, Chakravarthy U, Rosenfeld PJ, Holz FG (2020). Consensus nomenclature for reporting neovascular age-related Macular Degeneration Data: Consensus on Neovascular Age-Related Macular Degeneration nomenclature Study Group. Ophthalmology.

[16] Rajesh B, Kaur A, Giridhar A, Gopalakrishnan M (2017). Vacuole” sign adjacent to retinal pigment epithelial defects on Spectral Domain Optical Coherence Tomography in Central Serous Chorioretinopathy Associated with Subretinal Fibrin. Retina.

[17] Baek J, Kook L, Lee WK (2019). Choriocapillaris Flow Impairments in Association with Pachyvessel in Early Stages of Pachychoroid. Sci Rep.

[18] Schuman SG, Koreishi AF, Farsiu S, Jung SH, Izatt JA, Toth CA (2009). Photoreceptor layer thinning over drusen in eyes with age-related macular degeneration imaged in vivo with spectral-domain optical coherence tomography. Ophthalmology.

[19] Deak GG, Bolz M, Kriechbaum K, Prager S, Mylonas G, Scholda C, Schmidt-Erfurth U, Diabetic Retinopathy Research Group V (2010). Effect of retinal photocoagulation on intraretinal lipid exudates in diabetic macular edema documented by optical coherence tomography. Ophthalmology.

[20] Akagi-Kurashige Y, Tsujikawa A, Oishi A, Ooto S, Yamashiro K, Tamura H, Nakata I, Ueda-Arakawa N, Yoshimura N (2012). Relationship between retinal morphological findings and visual function in age-related macular degeneration. Graefes Arch Clin Exp Ophthalmol.

[21] Ogino K, Murakami T, Tsujikawa A, Miyamoto K, Sakamoto A, Ota M, Yoshimura N (2012). Characteristics of optical coherence tomographic hyperreflective foci in retinal vein occlusion. Retina.

[22] Coscas G, De Benedetto U, Coscas F, Li Calzi CI, Vismara S, Roudot-Thoraval F, Bandello F, Souied E (2013). Hyperreflective dots: a new spectral-domain optical coherence tomography entity for follow-up and prognosis in exudative age-related macular degeneration. Ophthalmologica.

[23] Bolz M, Schmidt-Erfurth U, Deak G, Mylonas G, Kriechbaum K, Scholda C, Diabetic Retinopathy Research Group V (2009). Optical coherence tomographic hyperreflective foci: a morphologic sign of lipid extravasation in diabetic macular edema. Ophthalmology.

[24] Song IS, Shin YU, Lee BR (2012). Time-periodic characteristics in the morphology of idiopathic central serous chorioretinopathy evaluated by volume scan using spectral-domain optical coherence tomography. Am J Ophthalmol.

[25] Mones J, Garcia M, Biarnes M, Lakkaraju A, Ferraro L (2017). Drusen Ooze: a Novel Hypothesis in Geographic Atrophy. Ophthalmol Retina.

[26] Margolis R, Mukkamala SK, Jampol LM, Spaide RF, Ober MD, Sorenson JA, Gentile RC, Miller JA, Sherman J, Freund KB (2011). The expanded spectrum of focal choroidal excavation. Arch Ophthalmol.

[27] Hashimoto Y, Saito W, Noda K, Ishida S (2014). Acquired focal choroidal excavation associated with multiple evanescent white dot syndrome: observations at onset and a pathogenic hypothesis. BMC Ophthalmol.

[28] Kim H, Woo SJ, Kim YK, Lee SC, Lee CS (2015). Focal Choroidal Excavation in Multifocal Choroiditis and Punctate Inner Choroidopathy. Ophthalmology.

[29] Nishikawa Y, Fujinami K, Watanabe K, Noda T, Tsunoda K, Akiyama K (2014). Clinical course of focal choroidal excavation in Vogt-Koyanagi-Harada disease. Clin Ophthalmol.

[30] Shah VP, Shah SA, Mrejen S, Freund KB (2014). Subretinal hyperreflective exudation associated with neovascular age-related macular degeneration. Retina.

[31] Inoue M, Kadonosono K, Watanabe Y, Sato S, Kobayashi S, Yamane S, Ito R, Arakawa A (2010). Results of 1-year follow-up examinations after intravitreal bevacizumab administration for idiopathic choroidal neovascularization. Retina.

[32] Rosenfeld PJ, Brown DM, Heier JS, Boyer DS, Kaiser PK, Chung CY, Kim RY, Group MS (2006). Ranibizumab for neovascular age-related macular degeneration. N Engl J Med.

[33] Yannuzzi LA, Slakter JS, Gross NE, Spaide RF, Costa D, Huang SJ, Klancnik JM, Aizman A (2003). Indocyanine green angiography-guided photodynamic therapy for treatment of chronic central serous chorioretinopathy: a pilot study. Retina.

